# Identification of Mesenchymal Stem Cell Marker STRO-1 in Oral Reactive Lesions by Immunofluorescence Method

**Published:** 2015-09

**Authors:** Ali Dehghani Nazhvani, Seyed-Mojtaba Hosseini, Bita Tahoori, Maryam-Sadat Tavangar, Armin Attar

**Affiliations:** a Biomaterial Research Center, Dept. of Oral and Maxillofacial Pathology, School of Dentistry, Shiraz University of Medical Sciences, Shiraz, Iran.; b Cell and Molecular Research Club, Shiraz University of Medical Sciences, Shiraz, Iran.; c Students' Research Committee, School of Dentistry, International Branch, Shiraz University of Medical Sciences, Shiraz, Iran.; d Biomaterial Research Center, Dept. of Operative Dentistry, School of Dentistry, Shiraz University of Medical Sciences, Shiraz, Iran.

**Keywords:** Pyogenic Granuloma, Peripheral Ossifying Fibroma, Immunofluorescence Technique, Dental Mesenchymal Stem Cell, STRO-1

## Abstract

**Statement of the Problem:**

Stem cells are considered as new implement for tissue regeneration. Several niches in adult human body are colonized by multipotent stem cells but access to these potential reservoirs is often limited. Although human dental pulp stem cells isolated from healthy teeth have been extensively characterized, it is still unknown whether stem cells also exist in reactive lesions of oral cavity such as pyogenic granuloma and peripheral ossifying fibroma which are deliberated as inflammatory proliferation of different cell families.

**Purpose:**

The aim of this study was to explore for clues to see whether pyogenic granuloma or peripheral ossifying fibroma contain dental mesenchymal stem cell (DMSC).

**Materials and Method:**

Four pyogenic granuloma and four peripheral ossifying fibroma specimens were collected by excisional biopsy and preserved in PBS-EDTA at -86 °C. Then we cut them in 5µm diameter using Cryostat. Having been rinsed with PBS, the samples were stained with a primary mouse anti-human STRO-1 monoclonal IgM antibody. Afterward, a secondary goat anti-mouse IgM-FITC antibody was applied to detect STRO-1+ cells as probable stem cells by immunofluorescence technique.

**Results:**

Immunofluorescence microscopy revealed presence of STRO-1+ cells in these lesions, particularly localized on perivascular zone. The negative control group was not glowing.

**Conclusion:**

Based on these results, it was found that reactive lesions of pyogenic granuloma and peripheral ossifying fibroma have STRO-1 positive cells, which raises the possibility that these cells may be DMSCs.

## Introduction

Stem cells are promising tools for tissue regeneration. Their remarkable ability in proliferation and differentiation enables them to restore the structures of impaired tissues.(1) The need to generate variety of tissues as well as the restriction of differentiation potential of stem cells after birth (compared with embryonic stem cells) have led to discovery of various resources of adult stem cells.(2) Various places in human body are colonized by considerable number of these cells, however, since access to these areas by surgery may cause damages to them, surgery would be considered as restricting factor in using these cells. For example, collecting these cells from the CNS causes deficiencies and problems that put the benefit of using these cells into question. On the other side, the selected locations should have a high percentage of stem cells compared with the amount of removed tissue.

The application of adult stem cells (ASCs) in tissue engineering is not followed by legal and ethical issues. Recently, most of ASCs are multipotent mesenchymal stromal cells (MSCs)(2) that have the capability of being transformed to different cells such as osteoblasts,(3) hepatocytes,(4) neurons,(5) adipocytes, cementoblasts, odontoblasts,(6) and cardiomyocytes.(7) These cells can make specific lesions heal through secretion of anti-inflammatory and nutrient materials.

The major source of ASCs is the bone marrow; however, they can be obtained from other reservoirs such as adipose tissue(1) and dental pulp.(8-10) The importance of using non-neuronal somatic stem cells to generate other cells rests upon the fact that these cells can be obtained in high scale from the individual’s different tissues of body and it totally resolves the ethical issues and problems such as unavailability and transplant rejection.

Dental pulp and its supportive tissues are derived from a sort of tissue called ectomesenchyme, which is produced by interaction of neural crest cells and mesenchyme in embryonic period. Hence, dental stem cells are likely to have properties identical to mesenchymal stem cells such as those of bone marrow. Despite the similarities, these two cell groups have some differences. For instance, stem cells of dental tissue are more differentiated, undergo fewer changes, and can help more in development and progression of odontogenic rather than osteogenic differentiation.(11) In recent years, mesenchymal stem cells with high proliferative ability have been isolated from dental ectomesenchymal tissues such as periodontal ligament stem cells (PLSCs)(12) and dental pulp stem cells (DPSCs).(8-9) Moreover, based on a study, the DPSCs have been detected in hyperplastic pulpitis (pulp polyp),(13) since pulp polyp is characterized as a reactive lesion of oral cavity; it is likely to happen to other reactive lesions of oral cavity. Pyogenic granuloma is one of these lesions that are histopathologically similar to pulp polyp. Peripheral ossifying fibroma is another reactive lesion of oral cavity with a similar pathogenesis. To remove these lesions completely, both lesions are treated by excisional surgery. STRO-1 is a cell marker present on all clonogenic stromal precursors(14) and is most commonly used in stem cell researches as a reliable marker for mesenchymal stem cells.(15-16)

The aim of this study was to find clues on presence of dental mesenchymal stem cell (DMSCs) in these lesions, using an immunofluorescent technique to detect DMSC marker STRO-1. 

## Materials and Method

Eight patients with pyogenic granuloma and peripheral ossifying fibroma who had referred to Shiraz School of Dentistry were examined and their diagnosis was confirmed by an expert pathologist. Four pyogenic granulomas and four peripheral ossifying fibromas were recruited as samples in this study. After getting informed consent according to *deceleration of Helsinki*, the lesion were cut under sterile conditions, immersed in PBS-EDTA solution which was made up of 1% penicillin/ streptomycin (Invitrogen; USA) and 1% fungizone (Invitrogen; USA) and then placed in embedding matrix for frozen section (CellPath co.; UK) and was rapidly frozen in Cryostat. Then several 5-µ sections were cut from the middle of the samples and were placed on slides.

The slides were immersed in phosphate buffered saline (PBS) for 5-10 minutes. Then, they were placed in 1% peroxidase for 5 minutes to ensure the peroxide inside the tissue had gone away and that non-specific reactions have been prevented. The samples were again rinsed with PBS for 5 minutes and 10% horse or goat serum was poured on them to inhibit the non-specific immunoglobulins.

The primary antibody, STRO-1 (Santa Cruz Inc.; USA) with dilution of 1:50 remained on samples for 2 hours and the secondary antibody, goat anti–mouse IgM-FITC (Santa Cruz Inc., USA) with dilution of 1:50 remained on samples for 45 minutes. Having been rinsed with PBS, the samples were placed in a container wrapped in aluminum, and were transferred to be assessed through immunofluorescence microscopy (Oly-mpus BX41; UK). The primary antibody was replaced by PBS as negative control and sections of pulp tissues were used as positive control. 

## Results

**Figure 1:**
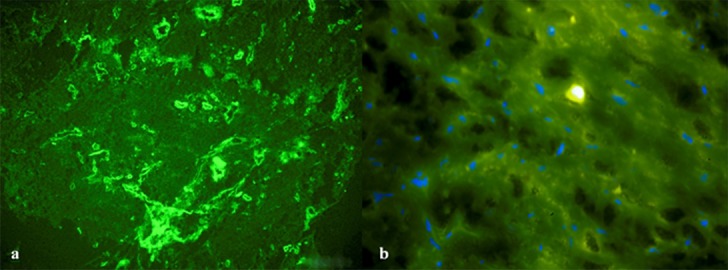
a: STRO-1+ cells in a sample of pyogenic granuloma under fluorescence microscope (200X) b: STRO-1+ cells in a sample of peripheral ossifying fibroma under fluorescence microscope (400X)

**Figure 2:**
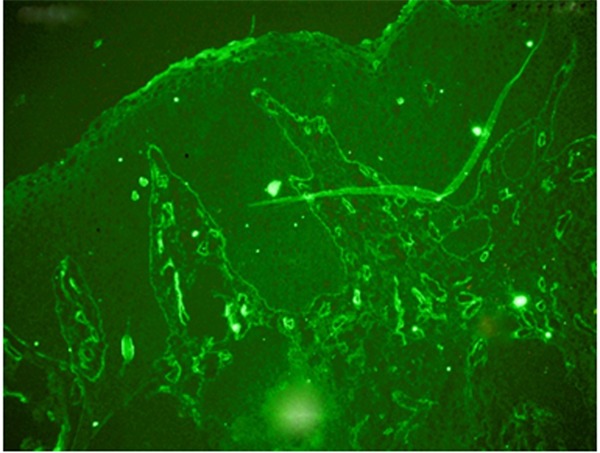
Presence of STRO-1+ cells in all areas from the subepithelium to the depth of tissue close to the walls and around blood vessels in a case of pyogenic granuloma under fluorescence microscope (100X)

In this study, four pyogenic granulomas and four peripheral ossifying fibromas were studied. Table 1 represents the patients’ demographic information. Assessing the STRO-1+ mesenchymal cells by using fluorescence microscope proved the presence of STRO-1+ stem cells in all samples (Figure 1). Concerning the location and dispersion, the identified STRO-1+ cells existed in all areas from the subepithelium to the depth of tissue, and the maximum accumulation was detected near the wall and around blood vessels (Figure 2).

**Table 1:** Patients’ demographic information and samples

**Tissue sources**	**Age**	**Sex**	**Location**
PG1*	10	F	Labial mucosa
PG2	17	F	Labial mucosa
PG3	25	F	Upper gingiva
PG4	24	F	Lower gingiva
POF1**	15	F	Upper gingiva
POF2	22	F	Upper gingiva
POF3	34	M	Upper gingiva
POF4	22	F	Lower gingiva

*Pyogenic Granuloma. **Peripheral Ossifying Fibroma.

It should be noted that the fluorescence intensity was lower in those slides that have been prepared sooner; and that negative control group showed no sign of presence of these cells. 

## Discussion

This study experienced the presence of STRO-1 positive cells within reactive oral pathosis. It may raise the hope of stem cell presence within these tissues which would be a new source of multipotent mesenchymal stem cells expecting them to be used for regenerating purposes in future, at least in dentistry. 

Markers of STRO-1 and CD146 are the most frequently used markers in studying stem cells.(14-19) They are known as markers of mesenchymal stem cells and have introduced the surrounding areas of veins as the most common areas for the presence of these cells. Although STRO-1 per se is a reliable marker for mesenchymal stem cells, the recent experiments have introduced it as a marker to be used for further phenotypic characterization and function of subset of mesenchymal stem cells such as rate of adherence and rate of proliferation.(18, 20-21)

Due to the high power of proliferation and differentiation, adult stem cells have been introduced as a promising tool in tissue engineering that are followed by less ethical and legal issues.(9, 22) The need for these cells in tissue regeneration as well as the restricted differentiation capability of adult stem cells in comparison with embryonic stem cells have resulted in exploration of different resources of ASCs, particularly MSCs. However, there still exists the need to find resources that are easily accessed and protect the patients against the harms of unwanted surgeries, as well as having more stem cells. Our efforts in this study raises the hope for identification of dental mesenchymal stem cells in lesions of pyogenic granuloma and peripheral ossifying fibroma called pyogenic granuloma stem cells (PGSCs) and peripheral ossifying fibroma stem cells (POFSCs). DMSCs are capable of forming dentin and pulp-like complex, and it makes the multipotent cells to be used as a perfect source for regenerating pulp and dental tissues.(23) Since the first researches, all types of dental mesenchymal stem cells have represented the ability to generate mineralized nodules with high levels of calcium when being placed in osteogenic culture medium,(15, 17, 24-25) and the researchers have tested and confirmed their differentiation ability.

Cryoprotection of cells and tissues, especially in repair processes, has significantly improved recently.(26) Up to now, only hematopoietic stem cells have been protected by cryoprotection(27-28) and since then, they have been successfully used in human transplantation.(29-30) Researchers have recently reported that after a long period of cryoprotection (almost 2 years), DPSCs and their differentiated osteoblasts had the ability to proliferate and generate woven bone *in vitro*; and *in vivo.* They had the ability to transform to mature lamellar bone just like new cells. It is not completely known how much the inflammation affects the differentiation potential of stem cells, however, it is recognized that mild inflammation improves the differentiation of odontoblasts and osteoblasts and increases the formation of matrix; while severe inflammation increases the apoptosis of stem cells.(31)

This study represented that pyogenic granuloma and peripheral ossifying fibroma had mesenchymal STRO-1+ stem cells. With respect to the great diversity of cells (such as inflammatory cells) in this reactive lesion, the properties of these cells like cultivation capability, phenotypic features, colony forming ability, the number of stem cells, their proliferative rapidity , and the differentiation potential of the approved categories must be studied and compared with healthy dental pulp stem cells. Also the right panel of cell surface antigen markers must be checked for them using flow cytometry so that their position would be clarified in the International Society for Cellular Therapy. 

## Conclusion

The results of this study revealed that reactive lesions of pyogenic granuloma and peripheral ossifying fibroma had mesenchymal STRO-1+ cells that would raise the possibility of the presence of stem cells within these tissues. Thus, since oral reactive lesions may serve as new possible stem cell reservoirs, their specific features and differentiation potentials should be evaluated. 
